# Microplastic in marine environment: reworking and optimisation of two analytical protocols for the extraction of microplastics from sediments and oysters

**DOI:** 10.1016/j.mex.2020.101116

**Published:** 2020-10-21

**Authors:** L. Rivoira, M. Castiglioni, S.M. Rodrigues, V. Freitas, M.C. Bruzzoniti, S. Ramos, C.M.R. Almeida

**Affiliations:** a Department of Chemistry, University of Torino, Via Pietro Giuria 7, 10125 Torino, Italy; b CIIMAR, Interdisciplinary Centre of Marine and Environmental Research, University of Porto, Terminal de Cruzeiros do Porto de Leixões, Av. General Norton de Matos s/n, 4450-208 Matosinhos, Portugal; c ICBAS – Institute of Biomedical Sciences Abel Salazar, University of Porto, R. Jorge de Viterbo Ferreira 228, 4050-313 Porto, Portugal

**Keywords:** Microplastics, Micropolymers, Marine sediments, Oysters, Contamination, Extraction procedures

## Abstract

Marine sediments and sessile biota (i.e. oysters) are nowadays recognised to be affected by microplastic (MP) pollution. NOAA proposes two distinct MP extraction protocols for sandy and bed sediments, which, however, were already demonstrated to suffer from many limitations. Conversely, to what concern oysters, works already published are usually time consuming, requiring a KOH 24–48 h oxidation step. The aim of this study is to show how iterative adaptation of the NOAA protocol allows to extract MPs, included PET, from marine sediments, regardless their characteristics. The method tested on PE-LD/PET/PA/PE-HD is based on density separation and oxidation treatments which were both carefully tuned, obtaining final recoveries higher than 85% for all the micropolymers (100% for PE and PA). Furthermore, a new protocol for the extraction of MPs from oysters was assessed, highlighting its efficacy (recoveries higher than 84% for all the plastics) and time-saving peculiarity. Finally, both protocols were successfully applied in the MPs extraction from real samples from Atlantic Ocean.

• The extraction of PE-LD/PET/PA/PE-HD was optimised in sediments (regardless their characteristics) and oysters.
• For sediments, density separation and oxidation procedures were carefully optimised.
• For oysters, oxidation times were reduced from 24 to 48 h to 1 h.

## Graphical abstract

**Figure:**
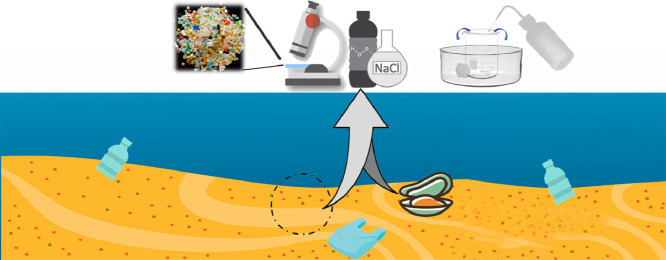

Specifications table

**Table:** 

Subject Area	Environmental Science
More specific subject area	*NA*
Method name	*Marine sediments and oysters microplastics extraction protocols*
Name and reference of original method	*J. Masura, J.E. Baker, G.D. Foster, C. Arthur, and C. Herring, Laboratory methods for the analysis of microplastics in the marine environment: recommendations for quantifying synthetic particles in waters and sediments, 2015.* *S. Rodrigues, C.M.R. Almeida, and S. Ramos, Adaptation of a laboratory protocol to quantity microplastics contamination in estuarine waters, MethodsX, 2019. 6: p. 740–749.*
Resource availability	*NA*

## Method details

Several procedures are nowadays investigated for the extraction of microplastics (MPs) from marine environment, with peculiarities typical of each investigated matrix (water, sediment, biota, etc.). Two main steps in common for all the extraction procedures are generally the ones that need adaptation to the investigated matrix: i) a density separation, where MPs are separated from their matrix, exploiting differences in terms of density between an extraction solution and MP polymers; ii) oxidation of organic matter, that float with separated MPs, thus making difficult MPs extraction and recognition.

Among the procedures already published, the NOAA (National Oceanic and Atmospheric Administration) protocol is one of the most applied [1]. It includes procedures focused on the analysis of MPs in water and sediments developed for polyethylene (PE), polypropylene (PP), polyvinyl chloride (PVC) and polyester (PEs), not considering polyethylene terephthalate (PET), which is one of the most detected plastic in the environment [2]. On the contrary, no procedures for biota are there described.

It was recently demonstrated how MPs, in particular fibres, can aggregate into clusters, thus becoming heavier and accumulating in marine sediments [3]. On the other hand, possible interactions of MPs with biota and in particular with bivalve organisms such as oysters, that are recognised to act like filters retaining marine pollutants, can occur [4], with possible MPs accumulation.

Moving from such considerations, efficient methods for the extraction of MPs from sediments and oysters are highly attractive.

Sediment composition greatly differs depending on the type of sediment. As also stated by NOAA [1], sediments can mainly be divided into two main categories, namely sand/sandy sediments, characterised by bigger particles, high amount of inorganic matter (i.e. silicates) and low amount of organic matter, and bed sediments, characterised by smaller particles and high amount of organic matter.

Differently, oysters are mainly characterised by water, but the high percentage of lipids (about 3% [5]) can highly interfere in the extraction of MPs [6], requiring a strong oxidation procedure that must not alter the polymers.

As previously mentioned, NOAA [1] has already developed two different procedures for the extraction of MPs from the sandy or bed sediments, mainly consisting of two sequential density separation steps and wet oxidation with hydrogen peroxide between them. Despite the important aim of developing standardised procedures for sample preparation and MPs identification, NOAA protocols were already demonstrated to suffer from many limitations, such as loss of MPs that are not correctly separated from the sand in the density separation [7]. Moreover, the corrected attribution of a sediment to its specific category (sandy/bed) and, therefore, to its appropriate extraction procedure, appears sometimes to be borderline. A unique procedure, that can be applied for the analysis of both types of sediments, is therefore hardly requested.

Differently, to what concern oysters, a commonly accepted protocol devoted to the extraction of MPs is missing. In literature, some works are focused on such matrix, but most of them are characterised by long oxidation processes that usually requires at least 24 h, only to remove lipids and organic matter [6].

Based on the above-mentioned considerations, and on expertise acquired by our research group on MPs extraction from estuarine waters [8], this work considers the extraction of a broad spectrum of MPs, including PET, from other seawater compartments (sediment and biota). More in detail, a unique iterative procedure is proposed for the extraction of MPs from sediment independently of its characteristics (sandy or bed type). Additionally, a quick and efficient protocol for the extraction of MPs from oysters is presented.

## MPs preparation

Polymers selected represent the most frequently detected MPs in the marine litter [6]: film plastic bags (Low-density Polyethylene (PE-LD); density 0.93 g/cm^3^), bottle cap particles (High-density Polyethylene (PE-HD, S); 1.00 g/cm^3^); bottle particles (Polyethylene terephthalate (PET); 1.38 g/cm^3^), fishing line fibres (polyamide, PA, commonly referred as nylon 6); 1.13 g/cm^3^) and microspheres deriving from personal care products (High-density Polyethylene (PE-HD, P); 1 mm, 1.00 g/cm^3^). Excepting the microspheres, all the remaining types of MPs were produced in laboratory from real samples (bags, bottle caps, bottles, fishing line, etc.) and sieved by 5 mm mesh-sieves to discard particles larger than 5 mm (falling outside the definition of MPs). Conversely, microspheres were commercially acquired from Cospheric (California, US) and used as received.

## Laboratory set-up

To avoid and monitor environmental contamination possibly present in laboratory, all steps were carried out inside a flow cabin during the entire proceeding and control blanks were run in parallel to samples. It should be mentioned that no MPs were detected in all blanks. Moreover, glass equipment rinsed with filtered deionised water was used. Work surfaces were cleaned with 70% ethanol solution and a lab coat and gloves were always worn. All the deionised water used during the protocols, including the water used in the solutions and the water used to rinse the material, was previously filtered (0.45 µm, mixed cellulose ethers), avoiding any type of external contamination.

NaCl and NaI salts, used to prepare saturated solutions, were from Sigma-Aldrich (Darmstadt, Germany) while ZnCl_2_ was from AlfaAesar, ThermoFisher Scientific (Kandel, Germany).

For MPs polymer spectra registration, a Perkin Elmer (Waltham, Massachusetts, U.S.) FT-IR Spectrum 2 instrument, coupled with attenuated total reflectance (ATR), was used. Stereomicroscope used for MPs counting was a Nikon SMZ800 instrument.

For each sample, MPs extraction recoveries (R%) were calculated by the number of MPs counted in laboratory fortified samples before and after the extraction procedure, according to the equation:

$$R\%=\frac{\text{MPs}\;\text{in}\;\text{pre}\;\text{extracted}\;\text{laboratory}\;\text{fortified}\;\text{sample}}{\text{MPs}\;\text{after}\;\text{the}\;\text{extraction}}$$

All extraction tests were repeated at least three times for each MP type.

## Extraction protocol for sediments

The method proposed for the extraction of MPs from sediments (regardless of whether sandy or bed sediments are investigated) is based on the following steps: 1) contamination of a laboratory fortified sample (only for simulating samples); 2) first density separation; 3) oxidation of organic matter; 4) second density separation; 5) analysis of MPs by means of a stereomicroscope. As detailed in the following paragraphs, steps 3) and 4) should be iteratively repeated for samples characterised by high amount of organic matter (e.g. bed sediments), when needed.

### Preparation of laboratory fortified sample

Before the optimisation of MPs separation, in order to correlate the method efficiency with sediment composition, both matrices were characterised in terms of organic matter (OM) content, obtaining 1% OM for sandy and 5% OM for bed sediments.

To what concern laboratory contamination of sediments, about 10 mg of each type of MP was weighted and polymer microparticles were singularly counted by means of a stereomicroscope. Subsequently, 100 g of sediments (previously sieved at 2 mm and dried at 90 °C overnight) were weighted and added to all the MPs, to obtain the laboratory fortified sample on which extraction procedure was developed.

### First density separation

The effect of saturated solutions in the density separation of MPs from sediments is highly discussed in literature [9]. Despite sodium chloride (NaCl) is the most used (density of saturated solution 2.16 g/cm^3^), other salts, such as sodium iodide (NaI, 3.67 g/cm^3^) or zinc chloride (ZnCl_2_, 2.91 g/cm^3^) are also suggested. Indeed, the higher the density of the salt solution, the higher the floating of MPs. As a drawback, high density solutions could also float other small particles (such as organic matter), thus increasing the presence of interference species. As a consequence, an intermediate density value for separation solutions should be found. In this regard, NaCl, NaI and ZnCl_2_ were investigated as saturated solutions to promote the density separation of MPs from sediments, having all of them higher densities than all the investigated polymers (see “MPs preparation” paragraph).

About 100 mL of each saturated solution were added to laboratory fortified samples, and vigorously stirred by means of a glass rod. It was immediately evident how the high amount of organic matter which was floating in NaI and ZnCl_2_ solution would have strongly affected MPs recoveries, in particular for bed sediments. Therefore, the density separation was continued only using NaCl saturated solution.

**Fig. 1:**
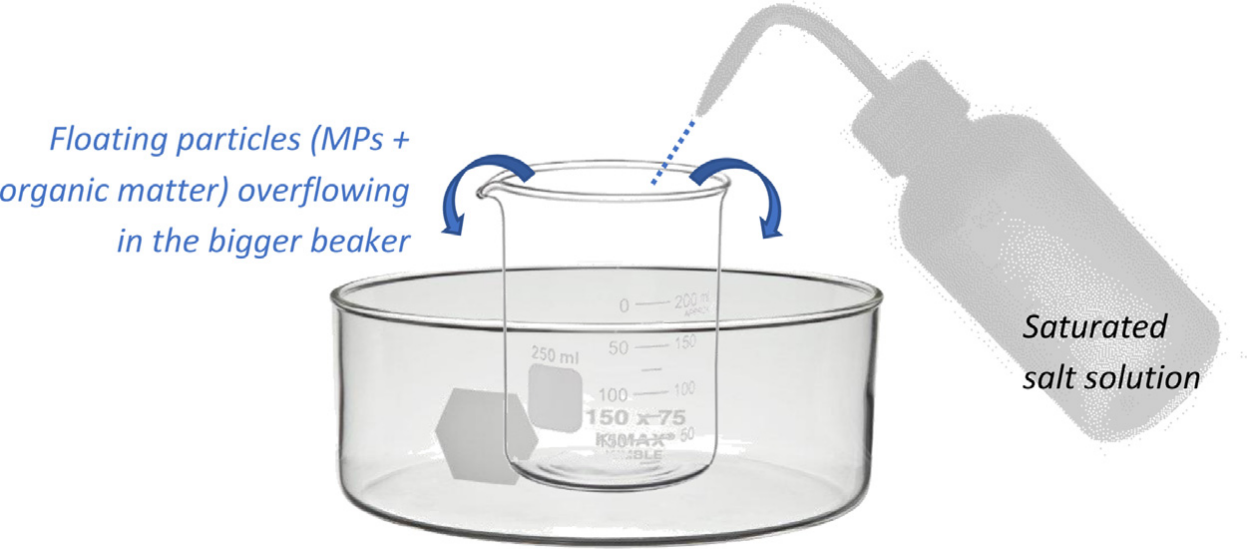
Lab-made glass equipment designed for the separation of floating MPs from sediment.

A separation time from 1 (sandy sediments) to 3 h (bed sediments) was required to settle all the small interfering particles; subsequently, floating particles (composed by both MPs and organic matter particles) were collected. Since NOAA does not specify a unique procedure to recover floating plastics, a laboratory-made glass equipment was arranged and hereafter described (Fig. 1).

Density separation was run in a smaller beaker (100–120 mL volume), which was placed in a bigger one (volume at least 3 times the smallest). Once the settlement of interfering particles occurred, the small beaker was filled very carefully with NaCl saturated solution, by means of a wash bottle, as long the solution overflowed in the bigger beaker, bringing MPs. In such a way, MPs were easily separated from sediment and heavier particles.

Differently to what is stated in NOAA protocol, a second extraction cycle (sediment stirring, sedimentation and MPs separation by NaCl overflow procedure) was performed, to better separate MPs still retained in the sediment.

Finally, to avoid possible MPs loss, NaCl saturated solution was used to clean the external surface of the smallest beaker, collecting in the bigger one all the particles potentially stick.

The so obtained suspension, was passed through a filter cloth (mesh < 0.03 mm, previously rinsed with deionised water and completely dried) and the bigger beaker was rinsed with a squirt bottle filled with NaCl saturated solution to transfer all residual solids to the filter cloth. To conclude the density separation procedure, the filter was cleaned with ultrapure water to remove all the salt traces and dried overnight (or up to complete dryness) in the oven at 90 °C.

As expected, the protocol improvement was demonstrated to increase the MPs extraction for both studied sediments (Table 1), in particular for PA (Nylon) fibres (30% increase for sandy sediments and 18% for bed sediments) and for PET particles (23% increase for sandy sediments and 20% for bed sediments), since they have the highest density among the tested polymers.

**Table 1:** MPs extraction recoveries, expressed as percentages, obtained after one or two density separation cycles and one oxidation treatment (mean values and standard deviation, *n* = 3).

	SANDY SEDIMENTS		BED SEDIMENTS	
	Recoveries [%] after one density separation cycle	Recoveries [%] after two density separation cycles	Recoveries [%] after one density separation cycle	Recoveries [%] after two density separation cycles
**PE-HD, S**	66 ± 1	80 ± 1	60 ± 1	75 ± 2
**PE-HD, P**	53 ± 1	73 ± 1	51 ± 1	72 ± 2
**PE-LD**	69 ± 2	88 ± 2	64 ± 1	76 ± 1
**PA, Nylon**	49 ± 2	79 ± 1	54 ± 1	73 ± 1
**PET**	45 ± 1	67 ± 1	46 ± 1	66 ± 1

The almost doubled extraction recovery obtained for PA (Nylon) fibres in sandy sediments in comparison to bed ones (30% vs 18%) is tentatively ascribed to a stronger adhesion of such MPs among bigger sand particles.

### Organic matter oxidation

As previously discussed, during the first density separation, small particles of light organic matter can float together with MPs, thus negatively affecting the separation of MPs. Indeed, organic matter can alter the extraction of MPs both promoting the formation of clusters with polymers (which can difficulty float in the subsequent second density separation) and/or making difficult the identification of plastics by optical microscope or spectroscopy techniques. An oxidation step is therefore necessary to remove such particles.

In our previous research, devoted to the development of an MPs extraction procedure from estuarine waters [8], the volume of a 0.05 M Fe(II) solution and of a 30% H_2_O_2_ solution to oxidize organic matter was deeply investigated, since the presence of iron activate the production of hydroxyl radicals (Fenton reaction [10]). In the above mentioned work, one oxidation cycle, performed on a hot plate at 75 °C, adding two consequent aliquots of 20 mL of oxidation solution, was shown to be enough to remove the interfering organic matter. However, when analysing sediment samples, the presence of high amounts of organic matter is usually faced, thus making not sufficient one cycle application. In the studied protocol, a consecutive application of oxidation cycles is therefore proposed, each cycle followed by a second density separation in a glass funnel (see the following paragraph).

In Table 2 recoveries obtained within the application of one, two or three oxidation cycles are presented, showing a significant difference among each other's for all the MPs (ANOVA test, confidence range 95%, *p* = 0.001). It should be mentioned that, when counting post extraction MPs to calculate the final extraction recovery, all the clusters with interfering species or plastic not fully identified were not considered.

**Table 2:** MPs extraction recoveries obtained after one, two or three oxidation cycles, for both sediments (mean values and standard deviation, *n* = 3). Recoveries were obtained after two density separation cycles.

	SANDY SEDIMENTS			BED SEDIMENTS		
	Recoveries [%] after one oxid. cycle	Recoveries [%] after two oxid. cycles	Recoveries [%] after three oxid. cycles	Recoveries [%] after one oxid. cycle	Recoveries [%] after two oxid. cycles	Recoveries [%] after three oxid. cycles
PE-HD, S	80 ± 1	92 ± 2	93 ± 1	75 ± 1	85 ± 1	97 ± 2
PE-HD, P	73 ± 1	99 ± 1	100 ± 2	72 ± 1	85 ± 1	96 ± 2
PE-LD	88 ± 1	94 ± 2	96 ± 1	76 ± 1	89 ± 2	99 ± 2
PA, Nylon	79 ± 2	81 ± 2	86 ± 3	73 ± 2	89 ± 2	100 ± 2
PET	67 ± 2	81 ± 2	85 ± 1	66 ± 1	78 ± 2	91 ± 1

For sandy sediments, which are characterised by a lower amount of organic matter, all MPs showed recoveries rates higher than 85% after the second oxidation cycle, thus suggesting that two cycles are enough to remove interfering particles. Differently, to what concern bed sediments, to completely remove organic species a third cycle was shown to be necessary.

Therefore, the obtained results suggest that two different methods to extract MPs from sediments (as suggested by NOAA) are not necessary. Conversely, an iterative application of the oxidation treatment to all the sediment samples (as proposed by our method) is enough to fully remove organic matter, with the number of oxidation cycles depending on the organic contamination of the treated sample.

**Fig. 2:**
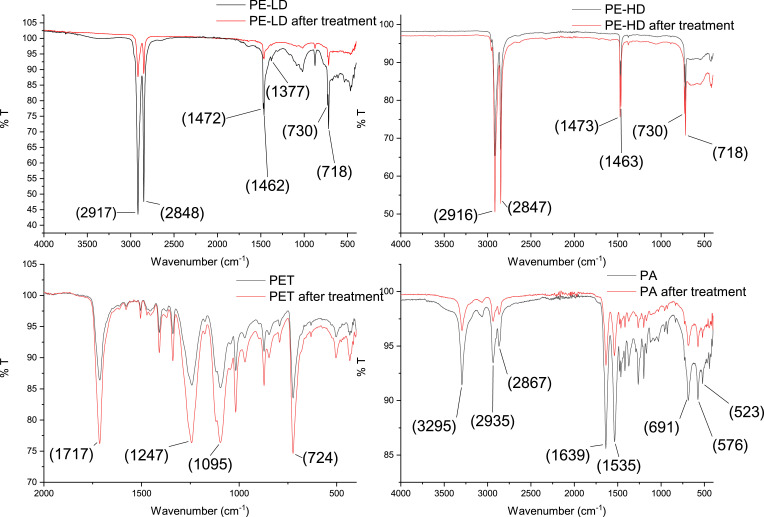
FT-IR analysis of MPs before and after oxidation treatment. Typical bands for each MP (expressed in cm^−1^) are also reported in brackets.

Finally, to check the integrity of MPs after the sequential application of the oxidation cycles, FT-IR analysis on both virgin and post extracted MPs were performed. As shown in Fig. 2, even after 3 oxidation cycles, the signals typical of each plastic polymer are still clearly distinguishable, thus demonstrating that no alteration of polymer chains occurred.

### Second density separation

**Fig. 3:**
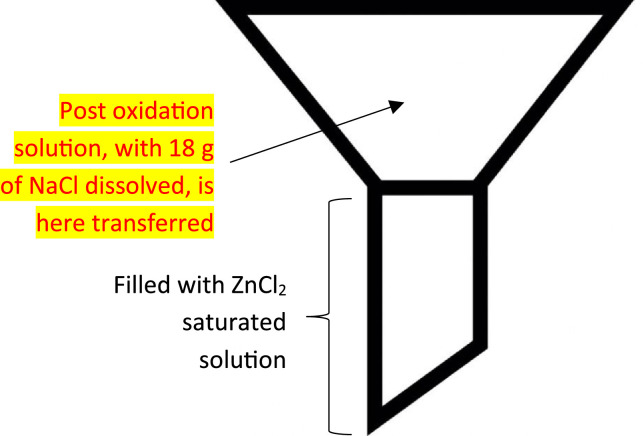
Scheme of the glass separation funnel, in which second density separation took place.

As stated in the previous paragraph, after each oxidation cycle (20 mL 0.05 M Fe(II) + 20 mL + 20 mL 30% H_2_O_2_ solutions, at 75 °C [8]) a second overnight density separation is required, in particular because it is frequent that inorganic particles, such as silica sand, are still present in the samples. Therefore, once the oxidative reaction took place, exactly 18 g of NaCl (6 g for each 20 mL of solution, with 20 mL of 0.05 M Fe(II) + 20 ml + 20 mL 30% H_2_O_2_ = 60 mL) were added and dissolved for 30 min in the samples (which was maintained at 75 °C), mixing the solution with a stirring magnet. Differently to what stated by NOAA protocol (that considers only the use of deionised water) [1], before transferring the saturated solution to the density separator funnel (a system made of a glass funnel, a latex tube at the bottom and a faucet to control the liquid flow), few mL of a ZnCl_2_ saturated solution are added to fill the final part of the funnel (Fig. 3).

With such implementation, sinking of heavier MPs, such as PET, is fully avoided.

Finally, the solution containing MPs could be transferred into the funnel, rinsing the beaker several times to quantitatively transfer all the particles. Even if in NOAA deionised water was proposed for this aim, in the presented protocol we strongly suggest to employ a NaCl saturated solution, using a washing bottle, to avoid a decrement in the density of the separation liquid and, therefore, the risk for MPs to sink. Subsequently, the funnel was covered with aluminium foil leaving potential interfering species, such as inorganic sand, to precipitate overnight.

Finally, after sedimentation time and before collecting MPs by means of the open flask filter already described in the previous paper [8], the faucet was opened, discarding the first mL of the solution (in which interfering sand particles or NaCl crystals are present). Extracted MPs, finally collected on the filter, were washed carefully with deionised water, dried in the oven at 90 °C and counted by means of a stereomicroscope.

### MPs overall recoveries

**Fig. 4:**
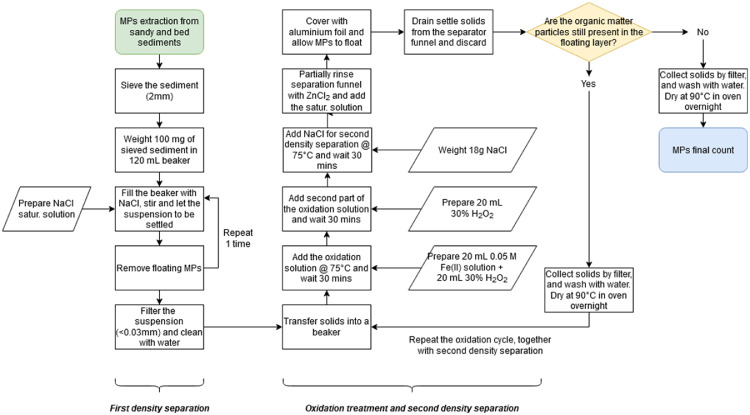
The overall scheme of the MPs extraction protocol in marine sediments.

A flowing diagram representing the entire extraction protocol is presented in Fig. 4, while the detailed step-by-step description is listed in the Supplementary material.

**Fig. 5:**
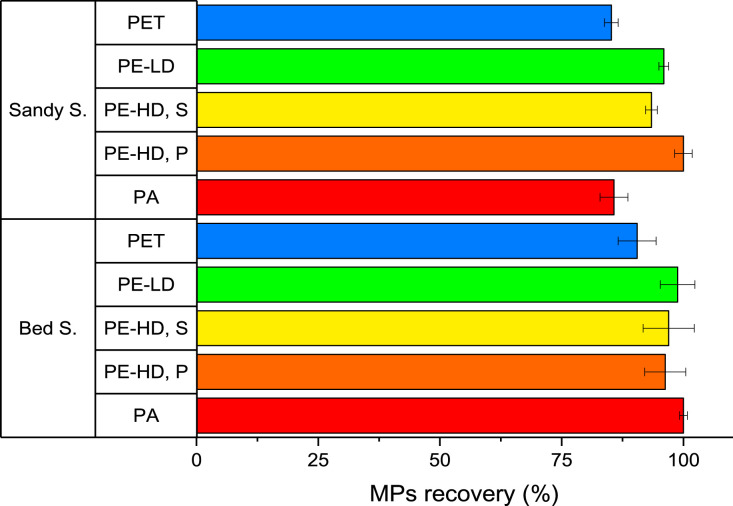
Overall MPs recoveries from sediments obtained with the optimised procedure (mean values and standard deviation, *n* = 3).

The overall extraction rate of the five tested microplastics using the modified protocol was evaluated, with the most of them recovered from the top of the density separator, regardless of the type of plastic polymer tested (Fig. 5).

Considering the overall improvements of the presented method, the repetition of oxidation cycles guaranteed the removal of most of the organic matter naturally present in sediments, avoiding the presence of organic particles floating in the density separator funnel and making possible to treat both types of sediments using the same procedure. Moreover, such oxidation procedure does not alter/degrade MPs polymer allowing their proper identification. Besides, thanks to the addition of ZnCl_2_ saturated solution in the bottom part of the separation funnel, no MPs sank, while all the remaining interfering and heavier sand particles were separated.

As shown in Fig. 4, recovery values were all higher than 85% for both studied sediments and all MPs types, despite their different composition in terms of organic matter content (1% OM for sandy and 5% OM for bed sediment). For both sediments, PET particles exhibited the lowest recoveries (around 85%), in agreement with their highest density in comparison to the other tested MPs. PA (nylon) fibres showed a similar recovery rate only for sandy sediments, while in bed ones extraction was quantitative. Such behaviour can be tentatively explained as fibres being more trapped by bigger sand particles than by the smallest ones, as also assessed when optimising the number of extraction cycles in the first density separation (see “First density separation” paragraph).

As concern the other MPs (PE-LD, PE-HD, S and PE-HD, P) they were almost quantitatively extracted from both sediments. Reproducibility was also confirmed, since relative standard deviations (*n* = 3) were lower than 3% for all the MPs.

When comparing obtained results with others already published, it is evident how by the proposed methods higher recoveries were achieved. As an example, Nuelle and co-workers showed PET recoveries from 14 to 68%, far lower that recoveries obtained in the presented manuscript. Low recoveries were also observed for PE in the same paper, as well as in the works of Coppock and co-workers and Rochman and co-workers [11].

Proposed results showed that no matter the type of MPs, a similar and almost quantitative extraction efficiency for high and low-density polyethylene, polyethylene terephthalate and polyamide can be obtained applying the same iterative protocol for all the type of marine sediments.

### Real sample application

The optimised protocol was successfully applied in the analysis of MPs from seven different sampled sediments, with organic matter content ranging from 0.4 to 0.9% (each analysis was performed in duplicate). Sediment samples were collected offshore Matosinhos coast (NW Portugal, of Matosinhos (41.20°N −8.74°W) near an artificial reef (a submarine wreck) at ca. 30 m depth. As shown in Table 3, all the sediments were characterised by the presence of several MPs.

**Table 3:** Results obtained after the application of the optimised protocol to seven real samples (two replicates per sample). Concentrations are expressed as n° fibres/100 mg of sediment.

	REAL SAMPLES: SEDIMENTS						
	*A*	*B*	*C*	*D*	*E*	*F*	*G*
*1st replicate*	50 fibres, no colour	28 fibres, no colour	10 fibres, no colour	48 fibres, no colour	26 fibres, no colour	60 fibres, no colour	42 fibres, no colour
		1 fibre, blue			1 fibre, blue		3 fibres, blue
		1 fibre, red				1 fibre, red	
*2nd replicate*	56 fibres, no colour	32 fibre, no colour	12 fibres, no colour	42 fibres, no colour	30 fibres, no colour	65 fibres, no colours	50 fibres, no colour
		1 fibre, blue			1 fibre, blue	2 fibre, blue	2 fibres, blue
						1 fibre, red	

In details, only fibres (both coloured and non-coloured) were detected, in agreement with recent studies showing how aggregation of such micropolymers can occur, followed by precipitation in sediments [3]. This area is known by intensive fishing activity and is considered a hotspot of lost gears in the NW Portuguese coast. So, the presence of MPs fibres could be probably ascribed to degradation of lost fishing nets that are found trapped in the wreck. However, MP polymer characterisation is still required to confirm this.

To what concern repeatability, it should be mentioned that MPs are discrete pollutants. Therefore, even if samples were fully homogenised before the application of extraction protocol, the presence of a different number of MPs in the two replicates (in particular for low detected polymers) should not be ascribed to low robustness of the method but to a small diversity of samples. Despite this premise, results presented in Table 3 showed how non-coloured fibres (the most abundant MPs) were detected in a similar concentration in both replicates.

## Extraction protocol for oysters

The method proposed for the extraction of MPs from oysters is based on the following steps: 1) contamination of oyster samples to have a laboratory fortified sample (only for simulating samples); 2) oxidation of lipids and organic matter; 3) density separation; 4) analysis of MPs by means of a stereomicroscope. As detailed in the following paragraphs, step 2) and 3) should be iteratively repeated for samples characterised by high amount of organic matter or fats, when needed.

### Laboratory fortified sample

Before the contamination with MPs, oysters (previously frozen at −20 °C) were measured (length and height of shell) and weighted. The oyster composition was measured before MPs separation, resulting in 2% lipidic content (wet weight), ca. 11% protein (wet weight) and ca. 78% moisture content. Such data are in good agreement with typical oyster composition [5], as stated in the introduction.

The shell was subsequently opened and the soft part of the animal was transferred into a 100 mL beaker. A few mL of NaCl saturated solution were used to wash the internal part of the shell in the beaker, to be sure that no MPs were still retained.

To simulate the MPs contamination, about 10 mg of each type of MP was weighted and polymer microparticles were singularly counted by means of a stereomicroscope prior to be added to the oyster soft tissue.

Finally, 25 mL of a 0.05 M Fe(II) solution were added in the beaker and its content was homogenised by means of blender an IKA (Staufen, Germany) T-10 ultrabasic TURRAX blender. After homogenisation, the upper foam formed is mainly due lipidic part of bivalves (Fig. S2 in the Supplementary material).

### Lipid and organic matter oxidation

As previously discussed, one of the most challenging aspect about MPs extraction from oysters is related to the presence of fats, that floating in aqueous solution, hamper the correct identification and count of all MPs potentially present. An effective oxidation treatment is therefore required.

In a recent work from Thiele and co-workers, where several oxidation treatments were tested and compared to remove organic matter [6], the most performing procedure proposed was a basic digestion with KOH, which requires 24–48 h to be effective. However, it is assessed that KOH can irreversibly degrade the MPs [12], on the other hand H_2_O_2_ is usually discarded since it can induce exothermic and explosive reactions, with the risk of losing MPS [6].

Based on the highly effective oxidation results obtained for estuarine waters [8] and sediments (see previous section about MPs extraction from sediments), in this work the oxidation properties of the 0.05 M Fe(II) solution together with 30% H_2_O_2_ were exploited, carefully tuning the volumes added.

In details, three initial different volumes of H_2_O_2_ solution were tested, namely 5, 10 and 15 mL, which were added to the homogenised solution (already containing 25 mL 0.05 M Fe(II) solution, see “Laboratory fortified sample” paragraph), heating the beaker at 75 °C. Whether 15 mL led to a high exothermic reaction (with the formation of foams that overflowed outside the beaker), 5 mL were shown not to be enough to activate the oxidation reaction. Conversely, the addition of 10 mL of 30% H_2_O_2_ was demonstrated to activate the production of hydroxyl radicals of Fenton reaction, avoiding any highly reactive reaction and loss of MPs.

Once the first oxidation reaction is finished, to complete the removal of lipids and foam, two subsequent 20 mL aliquots of 30% H_2_O_2_ solution were consecutively added and the solution was stirred at 75 °C for 1 h.

### Density separation

The solution obtained after the oxidation treatment was subject to a density separation (see “Second density separation” paragraph in sediment protocol). For that, 22.5 g of NaCl (6 g for each 20 mL, with 25 mL of 0.05 M Fe(II) + 10 ml +20 ml + 20 mL 30% H_2_O_2_ =75 mL) where added and dissolved in the samples (75 °C), mixing the solution with a stirring magnet for 30 min. The solution containing MPs was transferred into the separation funnel to settle overnight. Few mL of a ZnCl_2_ saturated solution were added to fill the lower part of the separation funnel, before transferring the solution.

After sedimentation time and before collecting MPs, the faucet was opened, discarding the first mL of the solution (in which interfering sand particles or NaCl crystals are present). The remaining solution is finally filtered by means of the open flask filter, washing carefully under deionised water the filter. The filter is then dried in the oven at 90 °C and MPs counted by means of a stereomicroscope.

Depending on the physical properties of the analysed oysters, a small portion of floating organic matter can be still present, thus making difficult the identification of MPs. In such occasion, another oxidation cycle should be performed, by subjecting the sample in the dry filter to the oxidation solution (20 mL 0.05 M Fe(II) + 20 mL + 20 mL 30% H_2_O_2_ at 75 °C), followed by the density separation step performed in the funnel.

### Overall recoveries

**Fig. 6:**
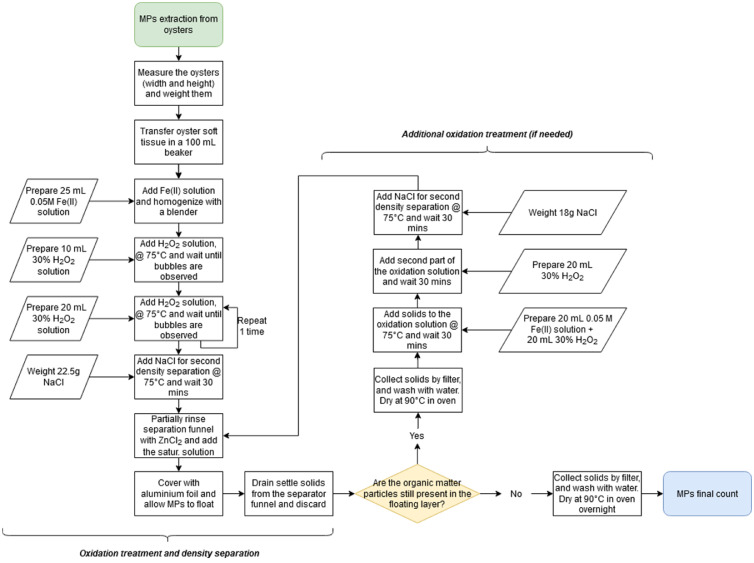
The overall scheme of the MPs extraction protocol in oysters.

A flowing diagram representing the entire extraction protocol is presented in Fig. 6, while the detailed step-by-step description is listed in the Supplementary material.

**Fig. 7:**
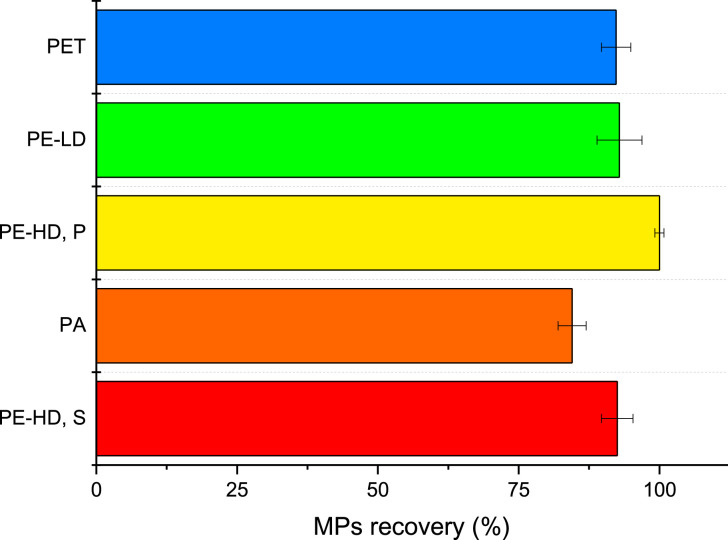
Overall MPs recoveries from oysters obtained with the optimised procedure (mean values and standard deviation, *n* = 3).

To evaluate the performances of the proposed protocol to extract MPs from oysters, the procedure was repeated three times analysing laboratory contaminated oysters, which were characterised by different dimensions and weight and calculating the recoveries of each MPs. Results are summarised in Fig. 7.

Results showed how all the MPs were efficiently extracted, with recovery yields ranging from 84.5% (PA fibres) to 100% (PE-HD, P). Average recovery for all MPs was 93%. Low standard deviations (lower than 4% for all MPs) confirmed the robustness of the proposed method. Moreover, none of the MPs was lost, nor broken by the homogenisation and oxidation steps.

It should be underlined that results obtained by the proposed protocol (1 h oxidation time) are far better than those presented by Thiele et al. [6], where PET and PE-LD MPs were recovered only at around 80% (with more than 5% standard deviation), after up to 48 h of oxidation.

### Real sample application

**Fig. 8:**
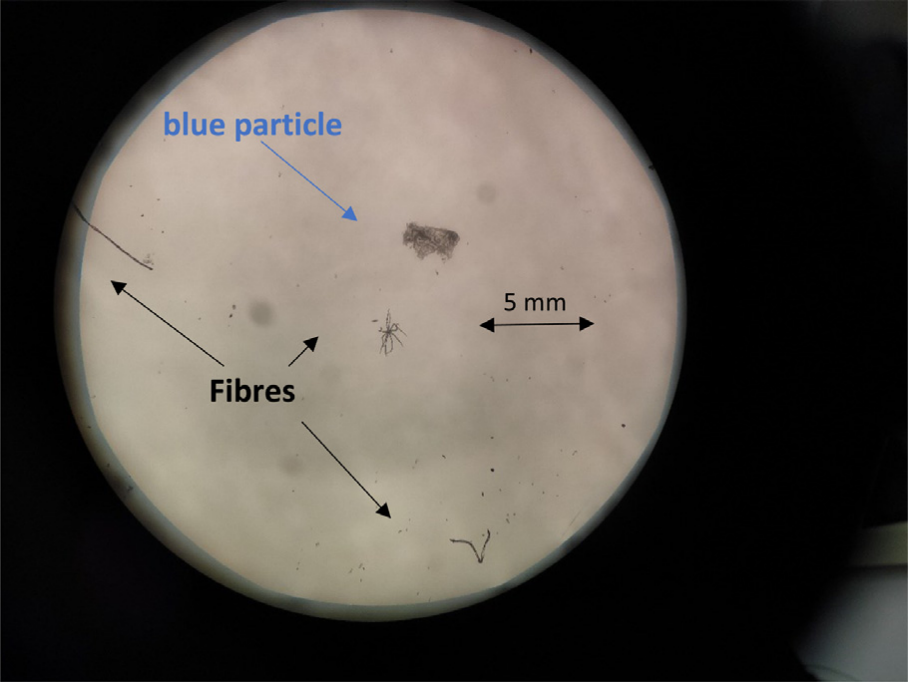
MPs extracted from an oyster. The picture was taken by stereomicroscope, in B/W mode, using a 10X magnification.

The optimised procedure was successfully applied for the extraction of MPs from three oyster samples (oysters collected in river Lima estuary, NW Portugal). As a result, few MPs fibres were detected. One blue particle was detected as well (Fig. 8). Future MP polymer characterisation is still required to confirm polymer type and possible MP source.

## Declaration of Competing Interest

The authors declare that they have no known competing financial interests or personal relationships that could have appeared to influence the work reported in this paper.

## Appendix Supplementary materials

Image, application 1
