# Supplementary material for: Microplastic in marine environment: reworking and optimisation of two analytical protocols for the extraction of microplastics from sediments and oysters
Source: MethodsX. 2020 Oct 21;7:101116. doi: 10.1016/j.mex.2020.101116 (PMC7596290; doi:10.1016/j.mex.2020.101116)
Supplement: Supplementary file 1 [file mmc1.docx]

**Supplementary material *and/or* Additional information:**

***SUPPLEMENTARY MATERIAL***

SEDIMENT PROTOCOL

A schematic view of the proposed method for the extraction of microplastics from all types of marine sediments is shown in Figure S1 and it is subsequently described in detail.

Sediment sieving

1st density separation

Oxidation treatment

2st density separation

Are organic matter particles still present
in the floating layer?

**YES**

**NO**

MPs collection and inspection

*Figure S1. Schematic representation of the MPs extraction method from marine sediments*

The method is hereafter detailed:

**1^st^ density separation**

1. Be sure to have already prepared a saturated NaCl solution (usually 1 L is required to treat 4 sediment samples). To obtain a saturated solution, 80g of NaCl should be added to each 250 mL of deionised water. Transfer part of the solution inside a washing bottle.
2. Pass the sediment sample in a sieve (mesh 2 mm) and collect the fraction which passed thought the sieve.
3. Weight the sediment, 100g in a 120 mL beaker
4. Add to the beaker the saturated NaCl solution to fill the beaker at least at ¾ of the volume
5. Stir carefully the sediment with a spoon (once withdrawn from the suspension, clean with some drops of saturated NaCl solution). Put the beaker inside a bigger one and fill the small beaker with saturated NaCl solution up to almost the highest border.
6. Let the suspension to be settled until the water phase is transparent (almost 1h for sand sediments and 3h for bed sediments)
7. Using the saturated NaCl washing bottle (or a glass pipette), carefully add the solution to the small beaker (avoiding any perturbation of the settled sediment) to let the floating layer overflowing the small beaker in the bigger one. If necessary, slightly recline the smaller beaker to help the overflow.
8. Stir again the sediment and let it settle as it did before. Repeat the same procedure to overflow the upper layer.
9. Manually remove the smaller beaker and clean its external surface inside the bigger one with saturated NaCl to remove MPs eventually stick.
10. Pass the solution from the bigger beaker through a filter cloth (mesh <0.03 mm previously rinsed with deionized water and completely dried)
11. Rinse the beaker thoroughly with saturated NaCl solution (used the washing bottle) to transfer all residual solids to the filter cloth
12. Clean the filter with deionized water to remove salt.
13. Dry it overnight (or up to complete dryness) in the oven at 90°C.

**Oxidation and second density separation**

1. Transfer solids collected in the filter cloth into the beaker using a spatula
2. Ensure all solids are transferred into the beaker
3. Turn on the hotplate to 75 °C
4. Add 20 mL of aqueous 0.05 M Fe(II) solution to the beaker containing the fraction of collected solids and then 20 mL of 30% H_2_O_2_ solution
5. Add a stir bar to the beaker and place them in the hotplate inside the fume hood
6. Heat to 75 °C for 30 minutes until gas bubbles are observed at the surface. If the reaction is too violent, remove the beaker from the hotplate until boiling subsides. If reaction appears to have the potential to overflow the beaker, add deionized water to slow the reaction.
7. When the reaction stops (no boiling) add another 20 mL of 30% H_2_O_2_ solution and heat to 75 °C letting it react as describe before (step before).
8. Add precisely 6 g of salt (NaCl) per 20 mL of sample to increase the density of the aqueous solution (ca. 5 M NaCl) (18 g of NaCl if you add 20 mL Fe(II) solution + 20 mL 30% H2O2 solution + 20 mL 30% H2O2 solution)
9. Heat mixture to 75 °C for 30 minutes or until the salt dissolves
10. Before transfer the saturated solution to the density separator funnel, add to the final part of the funnel with few mL of ZnCl_2_ saturated solution.
11. Rinse the saturated solution beaker with NaCl saturated solution to transfer all remaining solids to the density separator.
12. Cover loosely with aluminium foil
13. Allow microplastics to float overnight

**Collection of MPs**

1. Visually inspect settled solids for any microplastics. If any is found try to gently hit the separator, making sure all microplastics are floating above
2. Drain settled solids from the separator bottom and discard
3. Inspect if organic matter particles are still present in the floating layer:
   1. If yes, collect floating solids by passing the solution through the filter cloth immobilized in an open flask (rinsing the density separator several times with deionized water to transfer all solids to the filter cloth), wash them with deionized water and repeat the procedure of 2^nd^ day (oxidation and density separation)
   2. If not, proceed as subsequently described
4. Collect floating solids by passing the solution through the filter cloth immobilized in an open flask
5. Rinse the density separator several times with deionized water to transfer all solids to the filter cloth
6. Wash the filter cloth several times with running deionized water and then place the system with the filter cloth in a flask (covered with aluminium foil) with deionized water in the bottom for 5 minutes
7. Place the filter cloth in a petri dish and cover loosely with aluminium foil
8. Allow to dry for 24 hours at 90 °C

**MPs final count**

1. Collect all the remain material and microplastics from the filter cloth and count them by means of a stereomicroscope at 10-20X magnification.

OYSTER PROTOCOL


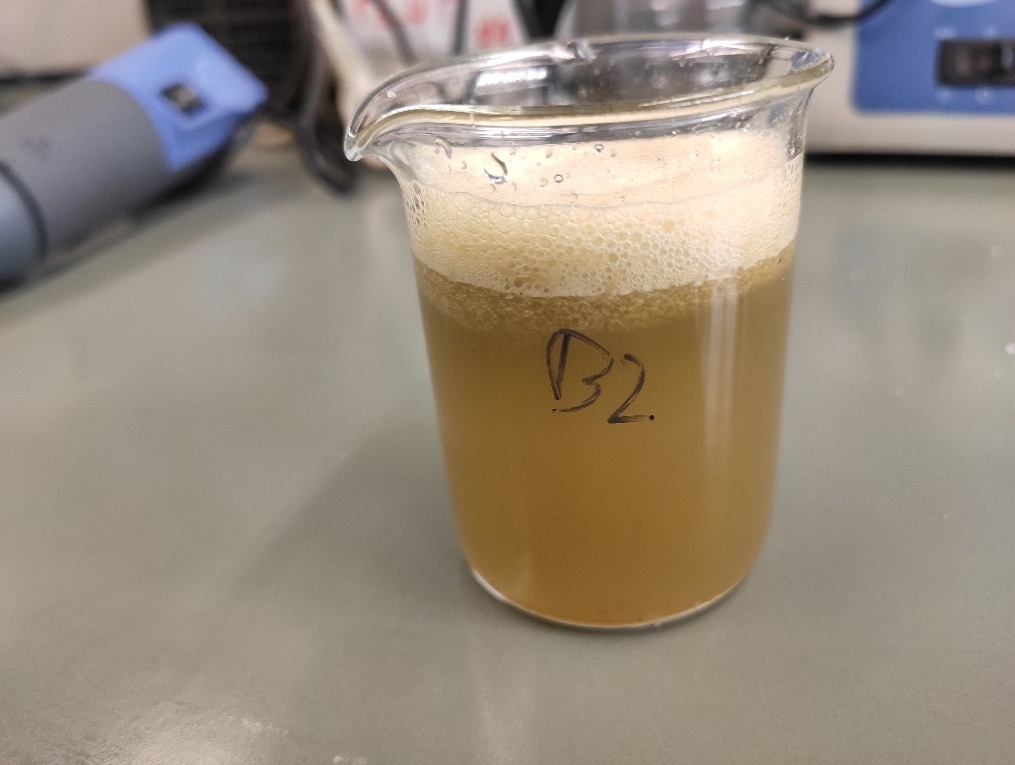


*Figure S2. Homogenized oyster soft tissue with 0.05 M Fe(II) solution. The upper foam is mainly due to lipidic composition of these bivalves.*

A schematic view of the proposed method for the extraction of microplastics from oysters is shown in Figure S3 and it is subsequently described in detail.

Measuring oyster dimensions

Oxidation treatment

1st density separation

Are organic matter particles still present
in the floating layer?

**YES**

**NO**

MPs collection and inspection

*Figure S3. Schematic representation of the MPs extraction method from oysters*

The method is hereafter detailed:

**Oxidation and density separation**

1. Be sure to have already prepared a saturated NaCl solution. To obtain a saturated solution, 80g of NaCl should be added to each 250 mL of deionised water. Transfer part of the solution inside a washing bottle.
2. Transfer oyster soft tissue inside a 100 mL beaker using a spatula. With few mL of NaCl saturated solution clean the internal part of the shell, to be sure that no MPs are still retained.
3. Add 25 mL of 0.05M Fe(II) solution to the beaker and homogenize the oyster by means of a blender.
4. Turn on the hotplate to 75 °C
5. Add 10 mL of 30% H_2_O_2_ solution
6. Add a stir bar to the beaker and place them in the hotplate inside the fume hood
7. Heat to 75 °C until gas bubbles are observed at the surface. If the reaction is too violent, remove the beaker from the hotplate until boiling subsides.
8. When the reaction stops (no boiling) add another 20 mL of 30% H_2_O_2_ solution and heat to 75 °C letting it react as described before. Repeat this step another time.
9. Add precisely 6 g of salt (NaCl) per 20 mL of sample to increase the density of the aqueous solution (ca. 5 M NaCl) (22.5 g of NaCl if you add 25 mL Fe(II) solution+ 10 mL 30% H_2_O_2_ solution + 20 mL 30% H_2_O_2_ solution + 20 mL 30% H_2_O_2_ solution)
10. Heat mixture to 75 °C for 30 minutes or until the salt dissolves
11. Before transfer the saturated solution to the density separator funnel, add to the final part of the funnel a few mL of ZnCl_2_ saturated solution.
12. Rinse the saturated solution beaker with NaCl saturated solution to transfer all remaining solids to the density separator.
13. Cover loosely with aluminium foil
14. Allow microplastics to float overnight

**Collection of MPs**

1. Visually inspect settled solids for any microplastics. If any is found try to gently hit the separator, making sure all microplastics are floating above
2. Drain settled solids from the separator bottom and discard
3. Inspect if organic matter particles or lipids are still present in the floating layer:
   1. If yes, collect floating solids by passing the solution through the filter cloth immobilized in an open flask (rinsing the density separator several times with deionized water to transfer all solids to the filter cloth), wash them with deionized water and repeat the procedure of 2^nd^ day (oxidation and density separation) using 20 mL 0.05 Fe(II) solution + 20 mL 30% H_2_O_2_ + 20 mL 30%H_2_O_2_.
   2. If not, proceed as subsequently described
4. Collect floating solids by passing the solution through the filter cloth immobilized in an open flask
5. Rinse the density separator several times with deionized water to transfer all solids to the filter cloth
6. Wash the filter cloth several times with running deionized water and then place the system with the filter cloth in a flask (covered with aluminium foil) with deionized water in the bottom for 5 minutes
7. Place the filter cloth in a petri dish and cover loosely with aluminium foil
8. Allow to dry for 24 hours at 90 °C

**MPs final count**

1. Collect all the remain material and microplastics from the filter cloth and count them with a stereomicroscope at 10-20X magnification.
